# The Antifungal Effect of Garlic Essential Oil on *Phytophthora nicotianae* and the Inhibitory Component Involved

**DOI:** 10.3390/biom9100632

**Published:** 2019-10-21

**Authors:** Yaochen Wang, Keke Wei, Xiaobin Han, Donglin Zhao, Yanfen Zheng, Jianmin Chao, Jianyu Gou, Fanyu Kong, Cheng-Sheng Zhang

**Affiliations:** 1 Marine Agriculture Research Center, Tobacco Research Institute of Chinese Academy of Agricultural Sciences, Qingdao 266101, China; wangyaochen2020@163.com (Y.W.); kekewei1995@163.com (K.W.); zhaodonglin@caas.cn (D.Z.); zhengyanfen@caas.cn (Y.Z.); caojianmin@caas.cn (J.C.); 2 Biological Organic Fertilizer Engineering Technology Center of China Tobacco, Zunyi 563100, China; hanxiaobin2011@163.com (X.H.); goujianyu1975@126.com (J.G.)

**Keywords:** garlic essential oil, diallyl disulfide, volatiles, *Phytophthora nicotianae*, biofumigant

## Abstract

This study explored the chemical compositions of garlic essential oil, the inhibitory activity of garlic essential oil and diallyl disulfide (DADS) against *Phytophthora nicotianae*, and the effects on mycelial plasma membrane permeability and *P. nicotianae* inhibition. In total, 29 compounds were detected in garlic essential oil, of which 26 were detected by gas chromatography‒mass spectrometry (GC-MS) and 21 by headspace solid-phase microextraction (HS-SPME) GC-MS. DADS (60.12% and 19.09%) and trisulfide di-2-propenyl (14.18% and 17.98%) were the major components identified by HS-SPME GC-MS and GC-MS analysis, respectively. Half-inhibitory concentration (Ec50, antagonism) and minimum inhibitory concentration (MIC, fumigation) of DADS against *P. nicotianae* were 150.83 μL/L and 20 μL/L, respectively, while Ec50 of garlic essential oil was 1108.25 μL/L. Mycelial membrane permeability gradually increased in a concentration-dependent manner, and cell death increased at 450 μL/L DADS. Furthermore, DADS treatment significantly reduced the incidence of tobacco black shank and the number of *P. nicotianae* pathogens in rhizosphere soil. DADS also promoted root development of tobacco seedlings at low concentrations, which was inhibited at high concentrations. Therefore, DADS may play an important role in the antifungal effect against *P. nicotianae* by destroying mycelial cell membrane integrity, causing an increase in cell membrane permeability, and leading to cell death.

## 1. Introduction

Tobacco black shank is a critical, global, soil-borne disease caused by *Phytophthora nicotianae* Breda de Haan [1] that has a high incidence and wide distribution range, and is highly destructive. In China, the epidemic area of the disease is more than 76,372 hectares, and the direct economic loss is over $1 billion annually. Currently, chemical fungicides such as metalaxyl‒mancozeb are the main control method, which can cause several issues, including high pesticide residues in tobacco leaves, environmental pollution, and pathogen resistance to fungicides [2]. Increasing concern about the environment and human health has prompted a drive to reduce fungicide use, and hence promote the development of other effective, ecofriendly control methods.

Essential oils (EOs) are aromatic and volatile oily liquids, extracted by hydrodistillation or supercritical fluid extraction from plants and spices, which are abundant in bioactive compounds and have antimicrobial and antioxidant properties [3,4,5,6]. Compounds such as terpenes and terpenoids have demonstrated antifungal activity and are easily degraded [7,8]. As a result, EOs are potential natural alternatives to reduce the negative impact of synthetic fungicides.

Crude garlic extract and certain chemical components have been proven to exhibit medicinal and bactericidal functions, and garlic use has gradually increased for agricultural disease control. Previous studies have shown that garlic extract has obvious inhibitory effects on *Botrytis cineria* Pers, *Ralstonia solanacearum*, and *P. nicotianae* [9,10,11,12]. Diallyl disulfide (DADS) is a colorless, oily liquid that has a garlic odor and is one of the most important components of garlic essential oil. It is used as an attractant and insecticide in feed and has also been shown to kill bacteria and inhibit soil nitrification [13,14]. Diallyl disulfide is a volatile organic substance and is considered to be a more stable compound than allicin, which is likely to decompose to DADS or other allelochemicals. Moreover, Gong et al. [15] speculated that sulfur-containing compounds from garlic straw are the main component inhibiting the growth of *Meloidogyne incognita*. Garlic extracts containing allicin or DADS, which are the primary sulfur compounds, were also reported to actively inhibit various pathogens such as *Fusarium oxysporum*, *Botrytis cinerea*, *Phytophthora capsici*, and *Verticillium dahliae* [16]. In addition, studies have shown that DADS can effectively control the occurrence of bean root rot, tomato root-knot nematode, and pepper blight, and is an organic sulfur fumigant with important application prospects [17,18]. 

However, there are few studies on the antimicrobial effects of DADS on crop pathogens, and its antimicrobial activity on *P. nicotianae* has not been reported. In this study, the chemical composition of garlic essential oil was examined, the inhibitory activity of garlic essential oil and DADS against *P. nicotianae* was investigated, and the effects of DADS on the plasma membrane permeability of mycelia were explored. These results provided insight into the application of essential oils as a potential fungicide for controlling *P. nicotianae* on tobacco and other crops.

## 2. Materials and Methods 

### 2.1. Materials

*Phytophthora nicotianae* (*Pn*) strain JM01, the pathogen, was isolated by Chengsheng Zhang and stored in our laboratory [19]. The tested tobacco variety was Xiaohuangjin 1025, which is susceptible to tobacco black shank disease. *P. nicotianae* was cultured in oat medium (OA) according to a method described previously by Han et al. [20].

Garlic essential oil and DADS were purchased from Benaco (Jingxi, China) and Macklin (Shanghai, China), respectively. Diallyl disulfide at 85% purity was dissolved in dimethyl sulfoxide (DMSO). Propidium iodide (PI) fluorescent dye was purchased from Thermo Fisher Invitrogen (Waltham, MA, USA), and the other conventional reagents (analytical grade) were all purchased from the China National Medicine Group (Tianjin, China).

### 2.2. Inhibition Test of Garlic Essential Oil against Phytophthora Nicotianae

For the antagonism test (Ec50), an OA medium plate containing garlic essential oil (diluted and dissolved in 2% DMSO) was prepared at final concentrations of 0, 800, 1000, 1200, 1400, and 1600 μL/L. Following, the colony diameter was observed and recorded after four days at 28 °C to calculate the Ec50. 

### 2.3. Gas Chromatography and Gas Chromatography–Mass Spectrometry Analysis

The essential oil was diluted with n-hexane-acetone (1:1, *v/v*) and analyzed simultaneously using gas chromatography (GC) and gas chromatography–mass spectrometry (GC/MS) systems [21,22]. The GC–MS analyses were conducted using an Agilent 7890-5975C GC–MSD system (Santa Clara, CA, USA) with a DB-5 MS fused-silica capillary column (30 m × 0.25 mm i. d (inside diameter, 0.25 μm film thickness). The injection volume was 1.0 μL in splitless mode with an inlet temperature of 250 °C, and helium was used as the carrier gas at a flow rate of 1.0 mL min^−1^. The analyses were carried out in programmed mode, the initial oven temperature was held at 45 °C for 2 min, ramped to 200 °C at 5 °C/min, held for 2 min, ramped to 320 °C at 15 °C/min, then held for 3 min. The transfer line, ion source, and quadrupole analyzer temperatures were 280 °C, 230 °C, and 150 °C, respectively. The ionization mode was the electron impact at 70 eV. The mass spectra plot was acquired using full scan monitoring mode with a mass scan range of m/z 35−450. The acceleration voltage was turned on after a solvent delay of 3 min. The GC analyses were performed using the Agilent 7890 GC system. The FID (flame ionization detector) temperature was set to 300 °C, and the same operational conditions were applied to a duplicate of the same column used in GC/MS analyses. The flow rate of carrier gas was adjusted to obtain the same retention times as the GC/MS analyses.

### 2.4. Headspace Solid Phase Microextraction Method for Sampling of Volatile Compounds

Headspace solid phase microextraction (HS-SPME) was chosen as the method of volatile compounds sampling [20]. The so-called sandwich type (DVB/CAR/PDMS, Supelco, Bellefonte, PA, USA) fiber was used for the absorption of essential oil volatile compounds. For the sampling of volatile compounds, the SPME fiber was inserted into the headspace of the sample vial containing 10 mg essential oil at room temperature for 20 min. When the sampling was completed, the SPME fiber was removed from the sample vial and immediately inserted into the GC injector at 250 °C for 5 min with a split ratio of 10:1. The used SPME fiber was conditioned at 250 °C for 5 min prior to the next sampling. Conditions of GC and GC/MSD are as mentioned above.

### 2.5. Inhibition Test of Diallyl Disulfide against Phytophthora Nicotianae

For the fumigation test (MIC), a mycelial disk from the edge of the *P. nicotianae* OA plate was transferred to the center of the solid OA medium. Following, DADS was dropped in the lid of the culture dish at 1, 2, 3, 4, 5, 6, 7, and 8 μL. Subsequently, the colony diameter was observed and recorded after 4 d. The minimum inhibitory concentration (MIC) of DADS on *P. nicotianae* was the concentration at total growth inhibition.

In the antagonism test (Ec50), an OA medium plate containing DADS (diluted and dissolved in 2% DMSO) was prepared, and final concentrations of the drug were added to the plate at 0, 120, 160, 200, 240, 280, or 320 μL/L. The mycelial disk from the edge of the *P. nicotianae* OA plate was transferred to the center of the solid OA medium and cultured for 4 d at 28 °C. Afterwards, the colony diameter was observed and recorded to calculate the Ec50.

### 2.6. Cell Membrane Permeability

Cell membrane permeability was measured by the electrical conductivity (EC). Five mycelial disks from the edge of the *P. nicotianae* OA plate were transferred into a liquid OA medium and cultured for two days at 28 °C and 175 rpm. Subsequently, 0.5 g of the mycelia was blended with 20 mL of 0, 300, or 450 μL/L DADS (diluted with 2% DMSO), and the EC values of the solutions were determined 0, 10, 20, 30, 60, 120, 180, and 240 min after treatment [23]. After 240 min, final conductivity was assessed by boiling mycelia for 5 min to totally kill the cells and release all electrolytes before cooling to room temperature [24,25]. The following formula was used to calculate the EC:

Relative Electrical Conductivity (%) = Conductivity at different time / Final conductivity × 100%.(1)

### 2.7. Propidium Iodide Fluorescence Assay of Phytophthora Nicotianae Mycelia

To evaluate DADS-induced cell membrane damage, a propidium iodide (PI) fluorescence assay was conducted [20,26]. *Phytophthora nicotianae* mycelia were harvested as follows. *P. nicotianae* mycelia were treated with 500 μL/L DADS for 24 h, and controls were prepared without DADS. Subsequently, the mycelia were filtered through double-layer gauze, and the mycelial disks were removed and washed three times with deionized water to complete the mycelial collection process. Next, 2 μmol/L PI (500 μL) fluorescent probe was added to the mycelia and stained for 30 min in the dark. Finally, the mycelia were washed three times with distilled water, observed under a fluorescence microscope, and photographed (excitation and emission wavelengths 535 and 615 nm, respectively).

### 2.8. Pot Experiment of Garlic Essential Oil and diallyl disulfide for Disease Prevention

To prepare the fungus millet, the millet was boiled with water until 60% flowering, followed by sterilization in a conical bottle and preservation. The *P. nicotianae* mycelial disk was selected using a pick needle, placed into a conical bottle, sealed, and cultured at 28 °C for two weeks [27].

For the fumigation test of DADS on *P. nicotianae* mycelia, 1 kg soil and 2–3 g fungus millets were mixed well with 50 mL diluted solution of DADS (500-times dilution, 1000-times dilution, diluted with 1% DMSO). Subsequently, the soil was sealed and fumigated with a plastic film (0.08 cm) for seven days, and tobacco seedlings of similar size were selected for transplanting. There were 15 replicates per treatment in this bioassay. Similarly, the fumigation test of garlic essential oil on *P. nicotianae* mycelia was conducted as mentioned above.

In the root irrigation test of DADS on *P. nicotianae* mycelia, 1 kg soil and 2–3 g fungus millets were mixed well, and tobacco seedlings of similar size were selected for transplanting. Subsequently, 50 mL diluted solution of DADS (500-times dilution, 1000-times dilution, diluted with 1% DMSO) was added to irrigate the tobacco root. There were 15 plants with three replicates per treatment in this bioassay. Similarly, the root irrigation test of garlic essential oil on *P. nicotianae* mycelia was conducted as mentioned above.

In the control treatment, 1 kg soil and 2–3 g fungus millets were mixed well with no DADS and garlic essential oil, and tobacco seedlings of similar size were selected for transplanting. There were 15 replicates per treatment in this bioassay. 

After seven days, the rhizosphere soil of tobacco seedlings was collected and frozen at ‒80 °C to prepare for quantitative real time polymerase chain reaction (RT-qPCR). Disease severity was recorded at 30 d, and the disease index was calculated according to Han et al. (2016) [27].

### 2.9. Dynamic Detection of Rhizosphere Pathogens by Real-Time Quantitative Polymerase Chain Reaction

Real-time quantitative PCR (RT-qPCR) was used to detect rhizosphere changes in *P. nicotianae*-infected soil after 10 days. The specific experimental methods are according to previous studies [28], with a few modifications. Briefly, RT-qPCR using DNA obtained rhizosphere soil, which was purified using the DNeasy^®^ PowerSoil^®^ Kit (Qiagen, Hilden, Germany) according to the manufacturer’s protocol. Real-time quantitative PCR amplification was performed using primers (SP: 5’-TGAAGAACGCTGCGAACTGC-3’, and AP: 5’-CTGACATCTCCTCCACCGACTA-3’) designed on the 18SrDNA gene sequence of *P. nicotianae* published in the NCBI database. The amplified fragment was 172 bp. Real-time quantitative PCR reactions were conducted in 20 μL reaction volumes containing 2.0 μL cDNA, 10.0 μL SYBR premix (TAKARA), 0.4 μL SP (10 μM/μL), 0.4 μL AP (10 μM/μL), and 7.2 μL ddH_2_O. The cycling protocol consisted of the following: 94 °C for a 5 min holding stage; 40 cycles of 94 °C for 20 s, 65 °C for 40 s, and 72 °C for 40 s.

The genomic DNA of *P. nicotianae* was diluted into six concentration gradients with three replicates per gradient with 10 dilutions, and the negative control was treated with sterile ultra-pure water. Quantitative PCR was used to collect fluorescence signals, confirm the amplification and melting curves, analyze the data by software, make standard curves, and generate standard curve equations.

### 2.10. Promoting Effect of Diallyl Disulfide

Tobacco seedlings of similar size were selected for testing after 14 days of growth. The loam and seedling substrate (1:1) were prepared and sterilized as testing soil. Subsequently, 20 mL diluted solution of DADS (100-, 200-, 500-, 1000-times dilutions; diluted with 1% DMSO, sonicated for 30 s) was added to irrigate the tobacco root. After seven days, seedling growth was observed, and the root length was measured. Each treatment was repeated three times.

### 2.11. Statistical Analysis

Analysis of variance (ANOVA), significance and linear regression analyses were conducted using Microsoft Office Excel 2003 and IBM SPSS Statistics version 19 (SPSS for Windows, Rel. 19.0.0. 2010, SPSS Inc., Chicago, IL, USA). Evaluations with *p* < 0.05 were considered significant for all tests.

## 3. Results and Discussion

### 3.1. Main Chemical Components of Garlic Essential Oil

The identification of essential oil components was based on the calculated retention indices (RI) and on comparisons of obtained mass spectra against reference compounds, available in the NIST (National Institute of Standards and Technology, Gaithersburg, MD, USA) library. RIs were determined in relation to a homologous series of *n*-alkanes (C7-C30) under the same operating conditions. The relative percentage amounts of the separated compounds were calculated from the integration of the peaks in FID chromatograms. 

**Figure 1:**
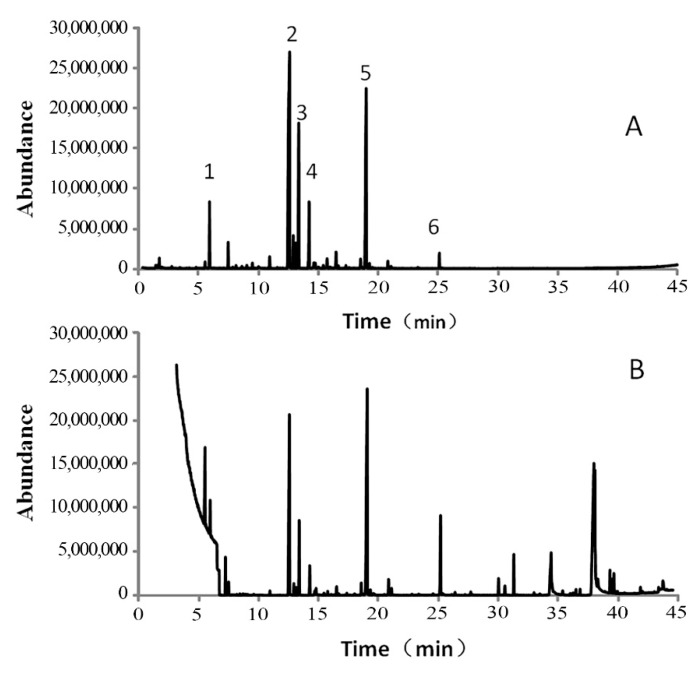
GC-MS total ion chromatograms of garlic essential oil. (**A**) Diluted with n-hexane; (**B**) volatile compounds sampling by HS-SPME method. 1, diallyl sulfide (DAS); 2, diallyl disulfide (DADS); 3, disulfide, dipropyl; 4, trisulfide, methyl 2-propenyl; 5, trisulfide, di-2-propenyl (DATS); 6, tetrasulfide, di-2-propenyl.

In total, 29 compounds were detected in garlic essential oil, of which 26 were detected by GC-MS and 21 by HS-SPME GC-MS analyses (Table 1 and Figure 1). Most of the detected compounds were sulfur-containing compounds, including cyclic sulfides and chain sulfides. Small amounts of terpenoids and fatty acid compounds were detected. Among them, DADS was the main volatile component (60.12%), followed by trisulfide di-2-propenyl (DATS) (14.18%), disulfide dipropyl (9.13%), and diallyl sulfide (DAS) (5.69%), which were detected by HS-SPME GC-MS. The compounds with the highest content, as detected by GC-MS analyses, were DADS (19.09%) and DATS (17.98%). Other compounds with high content included disulfide dimethyl, tetrasulfide di-2-propenyl, and some fatty acid compounds.

This shows that DADS and DATS were the major components identified using HS-SPME GC-MS and GC-MS analyses, in agreement with previous studies showing that the garlic essential oil consists of a variety of sulfides, such as DADS and diallyl trisulfide [29,30]. Mnayer et al. [31] reported the presence of DADS, DATS, DAS, allyl methyl, and trisulfide from the medicinal plants *Allium sativum*, *A. cepa*, *A. porrum*, *A. tuberosum*, *A. ascalonicum,* and *A. schoenoprasum*.

### 3.2. The Inhibitory Effect of Different Concentrations of Garlic Essential Oil and Diallyl Disulfide against Phytophthora Nicotianae

The Ec50 of garlic essential oil against to *P. nicotianae* was 1108.25 μL/L (y = 10.596x − 27.261, *R*^2^ = 0.984). 

**Figure 2:**
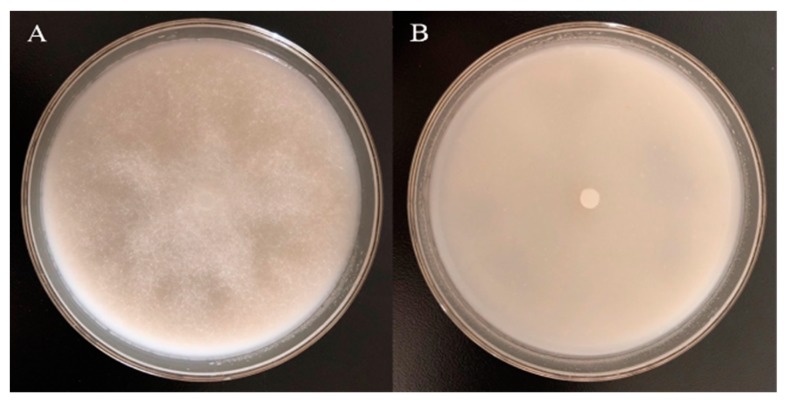
Fumigation effect of 20 μL/L DADS on *P. nicotianae*. (**A**) Control; (**B**) mycelia treated with 3 μL/dish (equivalent to fumigation concentration 20 μL/L) of DADS.

The chemical compound antifungal test on the main components of garlic essential oil showed that DADS was the main inhibitory active ingredient. Therefore, DADS was selected for further antifungal activity determination. DADS had a good inhibition effect on *P. nicotianae*. The Ec50 of DADS against to *P. nicotianae* was 150.83 μL/L (y = 5.736x − 7.9007, *R*^2^ = 0.9862). Afterwards, the MIC (fumigation effect) of DADS on *P. nicotianae* was 20 μL/L (3 μL/dish) (Figure 2), such that the inhibitory effect of DADS on *P. nicotianae* was greater than that of garlic essential oil. 

### 3.3. The Effect of Diallyl Disulfide on the Cell Membrane Permeability of Phytophthora Nicotianae

**Figure 3:**
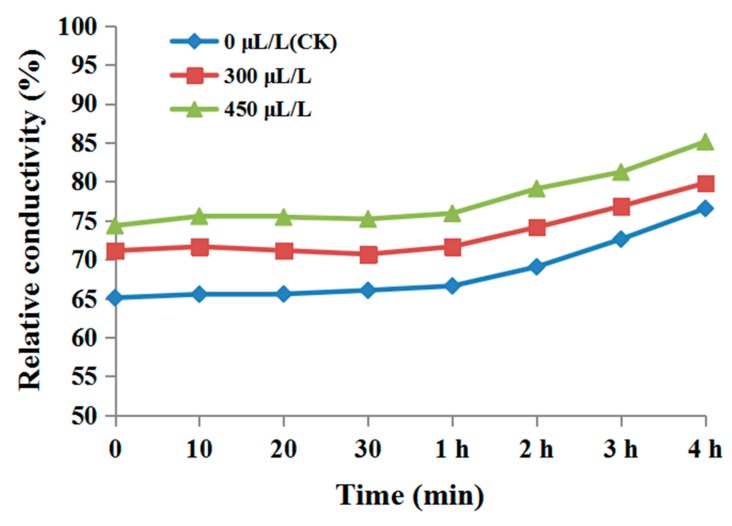
Effect of DADS on relative conductivity of *P. nicotianae* mycelia.

The cell membrane permeability change is shown in Figure 3. The relative electrical conductivity of the *P. nicotiana* mycelium solution treated with DADS was higher than that of control mycelia, and its conductivity increased over time and with increased DADS concentration. This indicated that DADS treatment destroyed the integrity of the mycelial cell membrane, rupturing the cell membrane and increasing intracellular electrolyte extravasation, thereby causing an increase in cell membrane permeability. This confirms that EOs severely interfere with the lipid layer of cell membranes and cause intracellular leakages, followed by cell lysis [32]. Zhang et al. [33] analyzed the protein leakage effect of the ethanol extract of *Mentha arvensis* on multidrug-resistant *Acinetobacter baumannii*. The intracellular protein released by the application of EO from *A. sativum* was significantly higher than that of intracellular protein released from *Acinetobacter baumannii*, which induced dose- and time-dependent rupture of the bacterial cell [34].

### 3.4. Propidium Iodide Fluorescence Assay of Phytophthora Nicotianae Mycelia

**Figure 4:**
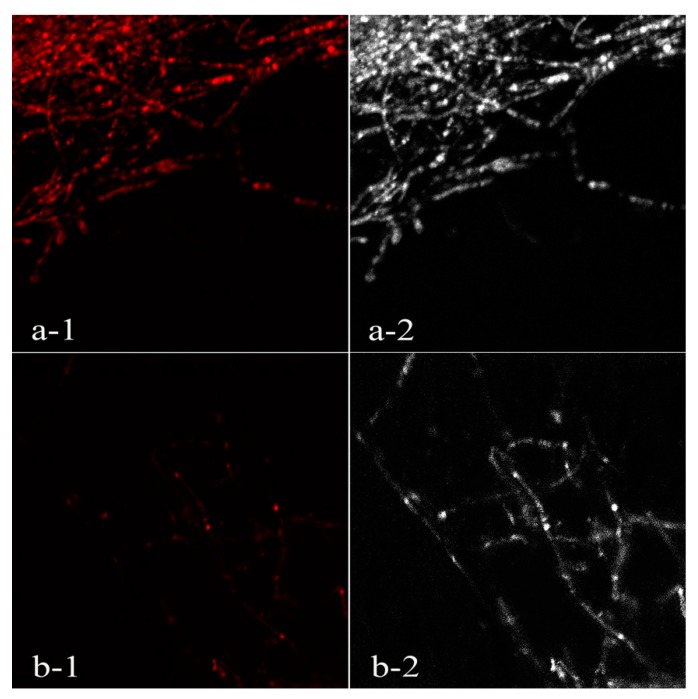
Effect of DADS on cell death of *P. nicotianae.* (**a-1**) Mycelia treated with 450 μL/L DADS under the laser field; (**a-2**) mycelia treated with 450 μL/L DADS under ordinary light field; (**b-1**) control under the laser field; (**b-2**) control under ordinary light field.

Compared to the control, 450 μL/L DADS-treated *P. nicotianae* mycelia were highly stained (Figure 4), indicating that DADS could destroy the cell membrane of *P. nicotiana* mycelia and induce apoptosis of mycelial cells.

Our studies were similar to others showing that the essential oil of *Chrysanthemum indicum* L. (EOC) significantly increased the EC values of *P. nicotianae* mycelia, and the integrity of mycelial cell membranes was destroyed by EOC [20]. Another study also confirmed that the cytoplasmic membrane damage and subsequent leakage of *Candida* species were caused by *Coriandrum sativum* essential oil [35].

### 3.5. Prevention of Phytophthora Nicotianae by Garlic Essential Oil and Diallyl Disulfide in Pot Experiment

As shown in Table 2, root irrigation and fumigation with GEO and DADS had a certain control effect against tobacco black shank (19.4‒59.7%). The control effect of DADS was higher than that of the same dosage of GEO, and the control effects were concentration-dependent. Consistent with the results of antifungal tests in vitro, fumigation treatment showed better disease control than root irrigation. These results indicated that DADS can serve as a fumigant for tobacco black control.

### 3.6. Dynamic Detection of Rhizosphere Pathogens by Real-Time Quantitative Polymerase Chain Reaction

The standard curve of y = ‒3.1199x + 39.913, *R*^2^ = 0.9977 was used for the dynamic detection of rhizosphere *P. nicotianae* by the RT-qPCR method. There was a significant difference between each treatment and the control (Table 3). Results suggested that DNA copies of *P. nicotianae* treated with a 500-times dilution of DADS were greater than the DNA copies treated with a 500-times dilution of garlic essential oil, which indicated that garlic essential oil diluted 500 times had a stronger inhibitory activity against *P. nicotianae* than DADS diluted 500-times in the root irrigation test. Furthermore, there were more DNA copies of *P. nicotianae* treated with a 500-times dilution of garlic than DNA copies treated with a 500-times dilution of DADS in the fumigation test. All drugs had a concentration effect, and DNA copies decreased significantly relative to the control.

Values are the means of three replicates ± SD. Values within the same column followed by different lowercase letters are significantly different (*p* < 0.05).

The results of the pot experiment showed that DADS treatment could significantly reduce the incidence of tobacco black shank and the number of *P. nicotianae* pathogens in rhizosphere soil. Studies have shown that DADS, as a biomimetic organic sulfur soil fumigant, has strong penetrability, rapid volatilization in the soil, and low requirements for soil water content. Therefore, the toxicity to plants is relatively low, and the detoxification period after application can be shortened accordingly [17].

### 3.7. Promoting Effect of Diallyl Disulfide

Results showed that the growth of tobacco seedlings treated with the 1000-times and 500-times dilutions of DADS showed no significant changes, and the root length increased significantly compared to the control. However, tobacco seedlings treated with the 200-times and 100-times dilutions of DADS were obviously weaker. Furthermore, the leaf growth was inhibited, wilting appeared, the root length was significantly longer and sparser compared to the control, and the root system was poorly developed (Table 4). As such, the results indicated that DADS promoted root development of tobacco seedlings at low concentrations, but inhibited development at high concentrations. This finding was consistent with that of Cheng et al. [36]. Kaili et al. [37] suggested that DADS had biological functions in plant root development and offered insight into the mode of action for this particular allelochemical of garlic. The effects were dose-dependent, with low concentrations having a promotional effect and high concentrations having an inhibitory effect on root growth. DADS actively influenced mitotic indexes and genes related to mitotic cell division, such as CDKA and CDKB. Nonetheless, it is bioactive in modulating functional plant hormones such as IAA, GA3, ABA, and CTK.

Values are the means of three replicates ± SD. Values within the same column followed by different lowercase letters are significantly different (*p* < 0.05).

## 4. Conclusions

Results indicated that garlic essential oil and its primary constituent had a good inhibitory effect on tobacco black disease caused by *P. nicotianae*. We found that DADS and trisulfide di-2-propenyl were the major components identified using the HS-SPME GC-MS and GC-MS analyses. Furthermore, our study found that the highest active ingredient of garlic essential oil was DADS, which had a strong fumigating effect on *P. nicotianae.* However, there were no studies available regarding the antifungal activities of DADS against *P. nicotianae*. Therefore, long-term, field-scale studies are needed to evaluate its fumigation effect.

## Figures and Tables

**Table 1:** Chemical composition of garlic essential oil (GC and GC-MS analysis).

Compounds	RT ^a^, min	Relative Amount (%)		Identification Method ^d^
		HS-SPME	SD ^b^	
Disulfide, dimethyl	3.43	0.05	Tr ^c^	MS
Diacetone alcohol	5.53	0.25	7	MS, RI
Diallyl sulfide (DAS)	5.9	5.69	1.78	MS, RI
Disulfide, methyl 2-propenyl	7.48	1.39	tr	MS, RI
Disulfide, methyl (1E) -1-propenyl	8.13	0.15	0.08	MS, RI
1,2-Dithiole	8.64	0.08	0.05	MS, RI
Dimethyl trisulfide	9.04	0.08	tr	MS, RI
α-Limonene	10.92	0.73	0.19	MS, RI
Diallyl disulfide (DADS)	12.62	60.12	19.09	MS, RI
1-Allyl-2-isopropyldisulfane	12.92	1.43	0.43	MS, RI
(*E*)-1-Allyl-2-(prop-1-en-1-yl) disulfane	13.1	1.07	0.32	MS, RI
Disulfide, dipropyl	13.38	9.13	2.95	MS, RI
Trisulfide, methyl 2-propenyl	14.25	2.24	0.86	MS, RI
4-Methyl-1,2,3-trithiolane	14.77	0.17	0.22	MS, RI
3-Vinyl-3,6-dihydro-1,2-dithiine	15.75	0.35	0.15	MS, RI
2-Vinyl-4H-1,3-dithiine	16.5	0.66	0.36	MS, RI
Carvone	17.31	0.12	0.11	MS, RI
Anethol	18.53	0.42	0.57	MS, RI
Trisulfide, di-2-propenyl (DATS)	19.02	14.18	17.98	MS, RI
1-Allyl-3-propyltrisulfane	19.29	0.14	0.17	MS, RI
5-Methyl-1,2,3,4-tetrathiane	20.83	0.14	0.33	MS, RI
Tetrasulfide, di-2-propenyl	25.12	0.45	3.85	MS, RI
1-Allyl-3-(2-(allylthio) propyl) trisulfane	31.23	tr	1.31	MS, RI
n-Hexadecanoic acid	34.37	tr	4.26	MS, RI
1-Allyl-3-(2-(allyldisulfanyl) propyl) trisulfane	36.41	tr	0.28	MS, RI
9,12-Octadecadienoic acid (*Z,Z*)-	37.92	tr	16.72	MS, RI
9-Octadecenoic acid, (*E*)-	38.01	tr	7.74	MS, RI
Octadecanoic acid	38.2	tr	0.42	MS, RI
Isopropyl linoleate	39.28	tr	0.64	MS, RI
β-Monolinolein	43.7	tr	0.61	MS, RI
Total identified (%)		99.03	88.47	

^a^ RT, retention time, ^b^ SD, solvent diluted, ^c^ tr Trace (<0.05%), ^d^ Identification method, MS: Comparison of mass spectra with mass spectral libraries, RI: Comparison to retention indices in libraries or literature.

**Table 2:** The control efficacy of the garlic essential oil and diallyl disulfide (DADS) treatments against tobacco black shank.

Treatment	Root Irrigation Effect		Fumigation Effect	
	Disease Index	Control Effect (%)	Disease Index	Control Effect (%)
GEO 1000 ^a^	66.67 ± 1.10 b	19.40 ± 0.53 d	48.15 ± 1.33 b	41.80 ± 0.265 c
GEO 500 ^b^	44.44 ± 2.42 d	46.35 ± 1.70 b	41.98 ± 1.39 c	49.27 ± 0.51 b
DADS 1000 ^c^	51.85 ± 2.02 c	37.36 ± 1.00 c	46.91 ± 1.85 bc	43.33 ± 0.93 c
DADS 500 ^d^	41.48 ± 1.21 d	49.87 ± 0.31 a	33.33 ± 2.42 d	59.80 ± 2.01 a
CK ^e^	82.72 ± 1.91 a	-	82.72 ± 1.91 a	-

^a^ 1000-times dilution of garlic essential oil diluted with 1% DMSO, ^b^ 500-times dilution of garlic essential oil diluted with 1% DMSO, ^c^ 1000-times dilution of DADS diluted with 1% DMSO, ^d^ 500-times dilution of DADS diluted with 1% DMSO, ^e^ control.

**Table 3:** DNA copies of *P. nicotianae* treated with garlic essential oil and DADS.

Treatment	Copies of Root Irrigation Effect	Copies of Fumigation Effect
GEO 1000 ^1^	558,251.04 ± 9385.42 b	15,531.74 ± 617.53 d
GEO 500 ^2^	38,276.60 ± 2752.51 d	1413.87 ± 98.33 d
DADS 1000 ^3^	238,512.00 ± 22,176.61 cd	4623.86 ± 246.49 d
DADS 500 ^4^	62,955.76 ± 80.94 d	752.19 ± 2.50 d
CK ^5^	9,234,399.25 ± 324,740.51 a	9,234,399.25 ± 324,740.51 a

^1^ 1000-times dilution of garlic essential oil diluted with 1% DMSO, ^2^ 500-times dilution of garlic essential oil diluted with 1% DMSO, ^3^ 1000-times dilution of DADS diluted with 1% DMSO, ^4^ 500-times dilution of DADS diluted with 1% DMSO, ^5^ control.

**Table 4:** Effects of different concentrations of DADS on tobacco root development.

Treatment	Root Length (mm)
CK ^1^	19.50 ± 0.764 d
DADS 1000 ^2^	27.33 ± 0.601 c
DADS 500 ^3^	27.83 ± 0.603 c
DADS 200 ^4^	41.50 ± 0.866 b
DADS 100 ^5^	44.83 ± 0.928 a

^1^ Control, ^2^ 1000-times dilution of DADS diluted with 1% DMSO, ^3^ 500-times dilution of DADS diluted with 1% DMSO, ^4^ 200-times dilution of DADS diluted with 1% DMSO, ^5^ 100-times dilution of DADS diluted with 1% DMSO.
